# Live‐cell FRET imaging‐based efficacy prediction of EGFR‐TKIs in EGFR L858R‐mutant NSCLC

**DOI:** 10.1002/ctm2.70878

**Published:** 2026-10-24

**Authors:** Xin Li, Lingao Dai, Pingshan Yang, Shuanghe Li, Chuan Peng, Shiyao Li, Beini Sun, Songwang Cai, Xiaoping Wang, Tongsheng Chen

**Affiliations:** ^1^ Department of Pain Management the First Affiliated Hospital of Jinan University Guangzhou China; ^2^ Department of Thoracic Surgery The First Affiliated Hospital of Jinan University Guangzhou China; ^3^ MOE Key Laboratory of Laser Life Science & Guangdong Provincial Key Laboratory of Laser Life Science College of Biophotonics South China Normal University Guangzhou China

**Keywords:** efficacy prediction, EGFR, FRET, NSCLC, precision oncology

## Abstract

**Objectives:**

Functional assays can assist individualised selection of treatment regimens by evaluating the ex vivo response of tumour cells to anticancer therapeutics. However, the two major strategies used to assess responses to targeted drugs, target‐based profiling and phenotypic profiling, have inherent limitations. Target‐based profiling does not directly capture downstream cell‐fate outcomes, whereas phenotypic profiling provides limited mechanistic information on drug action.

**Methods:**

Live‐cell fluorescence resonance energy transfer (FRET) microscopy was used to integrate the interaction between EGFR^L858R^ and GRB2 with cellular phenotypic features, thereby predicting the efficacy of EGFR‐tyrosine kinase inhibitors (TKIs) at the single‐cell level.

**Results:**

The target‐based drug score (*T*‐score), derived from the interaction between EGFR^L858R^ and GRB2 in living cells co‐expressing CFP‐EGFR^L858R^ and YFP‐GRB2, enabled evaluation of the on‐target activity of EGFR‐TKIs against EGFR^L858R^. The phenotype drug score (*P*‐score), constructed from cellular phenotype features, characterised the phenotypic responses induced by EGFR‐TKIs. The *TP*‐score, integrating the *T*‐ and *P*‐scores, accurately predicted the efficacy of EGFR‐TKIs. In living H3255 cells, the *TP*‐score of osimertinib (OSI) was higher than that of gefitinib (GEF). Notably, in H3255 cell‐derived xenograft (CDX) mouse models, OSI showed superior in vivo antitumour efficacy compared with GEF. Furthermore, live‐cell *TP* scoring revealed that primary tumour cells from a patient with NSCLC did not respond to OSI, a finding consistent with the absence of actionable EGFR mutations in this patient.

**Conclusions:**

This study demonstrates the clinical feasibility of predicting EGFR‐TKI efficacy by integrating EGFR^L858R^‐GRB2 interactions with cellular phenotypic features in single living cells.

## Graphical abstract

At the single‐cell level, live‐cell FRET microscopy was used to integrate the EGFR^L858R^‐GRB2 interaction with cellular phenotypic features to predict the efficacy of EGFR‐TKIs. The EGFR^L858R^‐GRB2 interaction‐based *T‐*score can evaluate the on‐target activity of EGFR‐TKIs against EGFR^L858R^. The *P*‐score can quantitatively characterise cellular phenotypic responses induced by EGFR‐TKIs. The *TP‐*score can accurately predict the efficacy of EGFR‐TKIs.

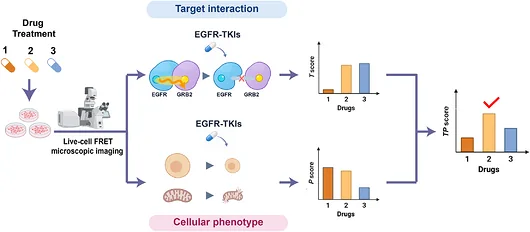

## 1 INTRODUCTION

A central challenge in clinical precision oncology is matching individual patients with the most effective therapeutic agents. Precision oncology currently relies primarily on genomic sequencing to identify patient‐specific genetic alterations and guide targeted therapy selection.^1^ However, the clinical benefit of genomics‐guided therapy remains limited. The rate of successful drug matching is approximately 10%, and the objective response rate (ORR) among matched patients generally does not exceed 20%.^2^ Patients with identical mutations often exhibit different sensitivities to targeted therapies.^3^ For EGFR L858R‐mutant non‐small cell lung cancer (NSCLC), more than ten first‐ to third‐generation EGFR‐TKIs have been approved.^4^ EGFR‐TKIs inhibit EGFR kinase activity and downstream signalling pathways to suppress tumour growth. First‐generation TKIs, such as gefitinib (GEF) and erlotinib, reversibly bind to the ATP‐binding pocket of EGFR, whereas second‐generation TKIs, such as afatinib (AFA) and dacomitinib, form irreversible covalent bonds with EGFR. Third‐generation TKIs, such as osimertinib (OSI), selectively and irreversibly target mutant EGFR harbouring sensitising mutations and the T790M resistance mutation.^5^ A network meta‐analysis of randomised controlled trials in advanced EGFR L858R‐mutant NSCLC found that no first‐line EGFR‐TKI demonstrated an overall survival (OS) advantage over other first‐line EGFR‐TKIs.^6^ However, Shimbu et al. reported substantial interindividual variability in sensitivity to different EGFR‐TKIs among patients with EGFR L858R‐mutant NSCLC.^7^ These findings suggest that population‐level evidence may be insufficient to identify the optimal therapy for individual patients.^8^

As a complementary approach to guiding cancer therapy,^9^, ^10^ ex vivo functional assays for targeted drugs primarily employ two drug screening strategies: assessing drug‐induced changes in target activity and phenotypic responses in tumour samples.^11^ Drug target profiling techniques, such as single‐molecule pull‐down and co‐immunoprecipitation (SMPC),^12^ kinase activity profiling using peptide arrays,^13^ and liquid chromatography‐tandem mass spectrometry (LC‐MS/MS) assays,^14^ frequently rely on the analysis of dead cancer cells^12^, ^13^, ^14^, ^15^ and often fail to reliably predict downstream biological effects.^14^ Using an LC‐MS/MS assay, Gosline et al. reported that FLT3 expression and phosphorylation did not correlate significantly with ex vivo cell viability responses to the FLT3 inhibitor quizartinib in samples from 38 patients with acute myeloid leukaemia (AML).^14^ Conversely, phenotypic profiling can capture drug‐induced cellular outcomes, but it cannot determine whether the observed responses are mediated by the intended drug target, particularly in the presence of off‐target effects.^16^, ^17^, ^18^, ^19^ Zhai et al. found that tumour cells from a patient with liver cancer exhibited more than 50% viability inhibition in response to the multi‐target TKI sorafenib using a digital microfluidic drug screening platform. However, subsequent whole‐exome sequencing revealed no mutations in sorafenib‐targeted genes.^19^

Live‐cell fluorescence resonance energy transfer (FRET) imaging and high‐content screening (HCS) have both been applied to drug screening. FRET imaging enables real‐time monitoring of protein–protein interactions (PPIs) in living cells and can be used to assess drug‐induced changes in target activity.^20^ In 1995, Lee et al. employed FRET to rapidly screen for novel HIV‐1 integrase inhibitors to facilitate antiviral agent development.^21^ In recent decades, FRET‐based screening methods have expanded in drug discovery to encompass additional compound classes,^22^ such as anticancer agents,^23^ G protein‐coupled receptor modulators,^24^ membrane‐potential modulators,^25^ antiparasitic drugs,^26^ and Ca^2+^‐signalling modulators.^27^ For clinical applications, Mizutani et al. developed a FRET‐based biosensor to predict the clinical efficacy of BCR‐ABL TKIs in patients with chronic myeloid leukaemia (CML) by measuring BCR‐ABL kinase activity in ex vivo bone marrow mononuclear cells.^28^, ^29^, ^30^ HCS captures multiparametric morphological features and identifies complex phenotypic alterations without prior assumptions, thereby characterising cellular responses to drug treatments.^31^ Initially developed in the mid‐1990s as a novel drug discovery tool,^32^ HCS has since been extensively applied in drug development, functional genomics, and fundamental biological research.^33^ Previous reports indicated that more than six commercial automated HCS microscopy platforms and more than 20 open‐source or commercial image‐analysis software packages were available.^34^, ^35^ In the last decade, the use of HCS in precision oncology has steadily expanded.^36^, ^37^, ^38^, ^39^ For example, Oh et al. employed HCS on tumour cells from 12 glioblastoma patients, identifying one patient whose tumour cells were sensitive to MET‐TKIs.^38^ Similarly, in a single‐arm, open‐label study, Kornauth et al. used single‐cell HCS to guide treatment decisions in patients with advanced, aggressive hematologic cancers. Among patients whose treatment was guided by HCS, 54% experienced more than a 1.3‐fold improvement in progression‐free survival (PFS) compared with prior therapy, and 40% achieved at least a threefold extension of absolute PFS relative to the expected median.^39^

Live‐cell FRET microscopy enables simultaneous analysis of EGFR^L858R^–GRB2 interactions and cellular phenotypic changes at the single‐cell level. EGFR primarily activates RAS–MAPK, PI3K–AKT, and JAK/STAT signalling, with GRB2 recruitment directly contributing to the RAS–MAPK and PI3K–AKT pathways.^40^ EGFR‐TKIs reduce the EGFR‐GRB2 interaction^41^, ^42^ and downstream signalling,^42^, ^43^ and these effects can alter mitochondrial morphology, promote apoptosis, and suppress proliferation.^43^, ^44^ Live‐cell FRET microscopy can quantify the EGFR‐TKI‐induced reduction in the EGFR‐GRB2 interaction^45^, ^46^ and can also reveal EGFR‐TKI‐induced morphological changes, such as changes in cell size and cell roundness.^47^

In this study, we integrated live‐cell FRET microscopy–based analyses of both EGFR^L858R^‐GRB2 interactions and cellular phenotypes to predict the efficacy of EGFR‐TKIs in H3255 cells (an EGFR L858R‐mutant NSCLC cell line) and the clinical efficacy of OSI in primary tumour cells from an NSCLC patient. We quantified EGFR^L858R^‐GRB2 interactions using live‐cell FRET imaging in cells co‐expressing CFP‐EGFR^L858R^ and YFP‐GRB2, deriving a target‐based drug score (*T*‐score). Additionally, phenotypic features extracted from bright‐field (BF) and mitochondrial (MITO) fluorescence images were used to calculate a phenotype‐based drug score (*P‐*score). The combined *T‐* and *P‐*scores formed an integrated *TP*‐score, which served as the final predictor of drug efficacy. In H3255 cells co‐expressing CFP‐EGFR^L858R^ and YFP‐GRB2, the *TP*‐score for OSI was higher than that for GEF, consistent with findings from H3255 CDX models. Furthermore, live‐cell *TP* scoring prospectively predicted primary insensitivity to OSI in a patient with NSCLC. Figure 1 presents the schematic workflow of live‐cell *TP* scoring, including sample preparation, data acquisition, and data analysis for drug efficacy evaluation.

**FIGURE 1:**
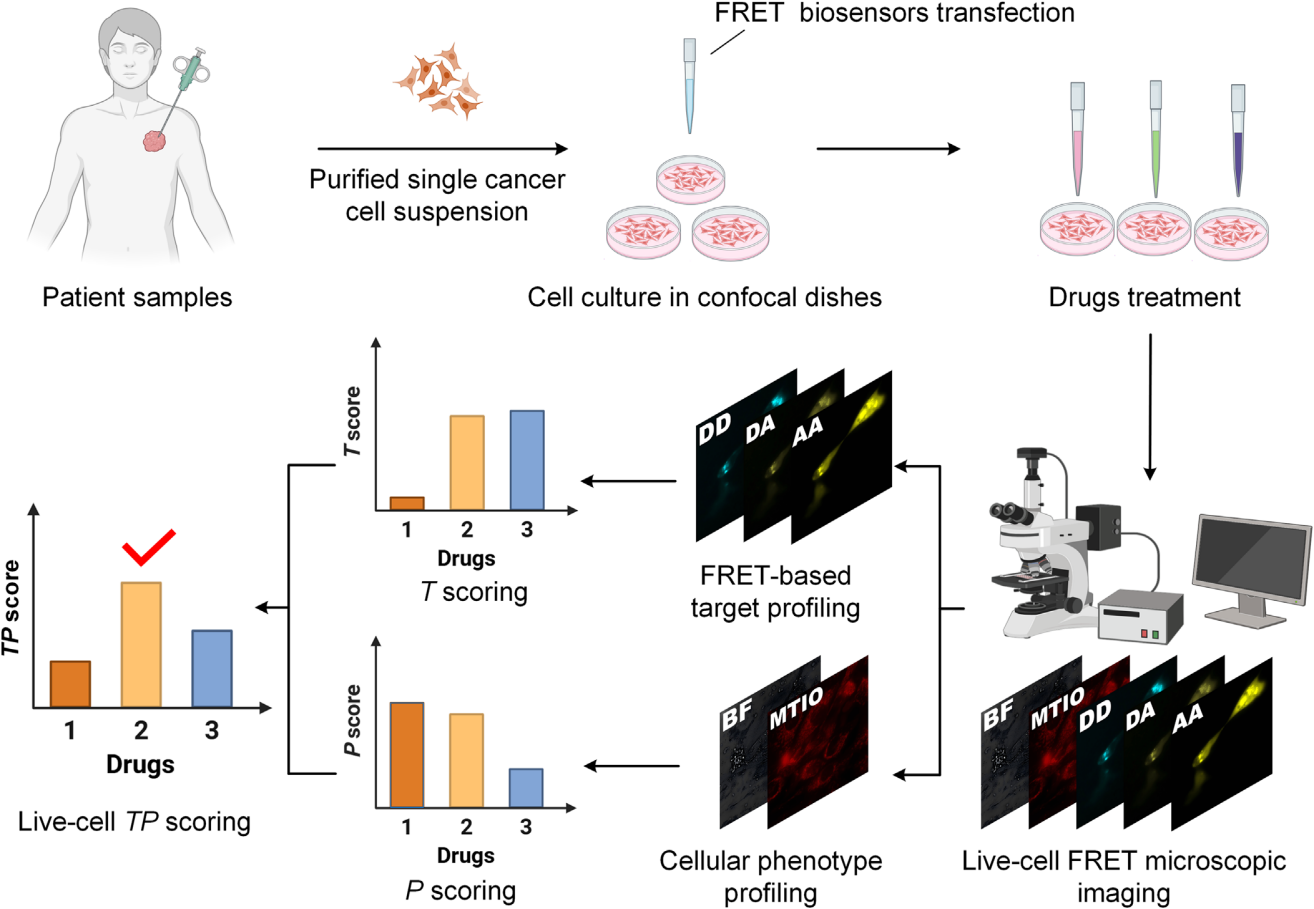
Schematic workflow of live‐cell *TP* scoring. Patient‐derived tumour samples were obtained via needle biopsy, pleural effusion sampling, or surgical resection. After dissociation and purification, tumour cells were cultured in 15‐mm confocal dishes. Cells were transfected with FRET biosensors (CFP‐EGFR^L858R^ and YFP‐GRB2 fluorescent proteins), incubated for 24 h (plasmid‐based biosensors) or 8 h (mRNA‐based biosensors), and subsequently treated with designated drugs. At 16 h post‐treatment, live‐cell FRET microscopy was performed to acquire three‐channel FRET images (DD, DA, AA), BF images, and MITO fluorescence images. Target‐based drug scores (*T*‐scores) and phenotype drug scores (*P*‐scores) were computed separately from these data and combined into integrated *TP*‐scores. Drugs with high *TP‐*scores, reflecting robust on‐target inhibition and strong phenotypic responses, were prioritised as candidate therapeutics for each patient.

## 2 MATERIALS AND METHODS

### 2.1 Materials

Fetal bovine serum (FBS), PBS, and RPMI 1640 medium were purchased from Gibco (Grand Island, NY, USA). The pCMV‐EGFR(human)‐L858R‐ECFP‐Neo (CFP‐EGFR^L858R^) plasmid was obtained from MiaoLing Plasmid Platform (Wuhan, China). The pCMV‐GRB2(human)‐EYFP‐Neo (YFP‐GRB2) plasmid was generated by homologous recombination of pCMV‐GRB2(human)‐3×HA‐Neo and pCMV‐Bad‐EYFP‐Neo. The pCMV‐GRB2(human)‐3×HA‐Neo plasmid was purchased from MiaoLing Plasmid Platform (Wuhan, China), and the pCMV‐Bad‐EYFP‐Neo plasmid was previously constructed in our laboratory.^20^ Lipofectamine™ 3000 transfection reagent, Lipofectamine™ MessengerMAX™ transfection reagent, and MitoTracker Deep Red 633 were acquired from Thermo Fisher Scientific (Waltham, MA, USA). GEF, AFA, OSI, alectinib (ALE), and gemcitabine (GEM) were purchased from MCE (New Jersey, USA). Cell Counting Kit‐8 (CCK‐8) was procured from Dojindo Laboratories (Kumamoto, Japan). A 4% paraformaldehyde solution was obtained from Biosharp (Beijing, China). Crystal violet staining solution was purchased from Beyotime (Shanghai, China). Matrigel was obtained from Mogengel Bio (Xiamen, China). The Tumor Dissociation Kit (human) was purchased from Miltenyi Biotec (Köln, Germany). The EasySep™ Human EpCAM Positive Selection Kit II was obtained from STEMCELL Technologies (Vancouver, British Columbia, Canada). The T7 High Yield RNA Synthesis Kit for Co‐transcription (low dsRNA) was purchased from Yeasen (Shanghai, China). The lentiCRISPR v2 plasmid was purchased from Qiyunbio (Guangzhou, China). The EGFR antibody was supplied by Cell Signaling Technology (Danvers, MA, USA). Servicebio (Wuhan, China) provided the β‐actin antibody.

### 2.2 Bioinformatics analyses

Protein–protein interaction networks were generated using the STRING v12.5 database (https://string‐db.org/)^48^ with the following settings: network type, physical subnetwork; meaning of network edges, confidence; active interaction sources, text mining, experiments, and databases; and minimum required interaction score, 0.900. KEGG pathway enrichment analyses were also performed using STRING (FDR ≤ 0.05). Correlation analyses of gene expression utilised GEPIA2 (http://gepia2.cancer‐pku.cn/)^49^ based on TCGA (https://www.cancer.gov/ccg/research/genome‐sequencing/tcga) lung adenocarcinoma (LUAD) data (*n* = 483), with Pearson's correlation coefficient. Gene expression levels in LUAD tumours and normal tissues from TCGA (tumour, *n* = 483; normal, *n* = 59) and GTEx (https://www.genome.gov/Funded‐Programs‐Projects/Genotype‐Tissue‐Expression‐Project, normal, *n* = 288) were analysed using GEPIA2. Differences in OS and PFS between high‐ and low‐expression groups were assessed using the ‘Expression & Survival’ module of GSCA (https://guolab.wchscu.cn/GSCA)^50^ based on TCGA LUAD data (tumour, *n* = 517; normal, *n* = 59; paired samples, *n* = 58). Correlations between gene expression and sensitivity to GEF and AFA across pan‐cancer data were also examined through GSCA, utilising the GDSC database (https://www.cancerrxgene.org/).

### 2.3 Cell culture

The NCI‐H3255 cell line was purchased from Meisen Chinese Tissue Culture Collection. Primary tumour cells were isolated from LUAD patient samples. Cells were maintained in RPMI 1640 medium supplemented with 15% FBS and cultured at 37°C in a humidified incubator containing 5% CO_2_.

### 2.4 Cell transfection

Cells were seeded in 15‐mm glass‐bottom dishes before transfection. H3255 cells were transfected with plasmids using Lipofectamine™ 3000 according to the manufacturer's instructions. For FRET experiments, CFP‐EGFR^L858R^ and YFP‐GRB2 plasmids were mixed at ratios of 1:1 or 1:2 and co‐transfected into the same dish.^51^ The total plasmid DNA per dish was 1000 ng. Primary tumour cells were transfected with mRNA using Lipofectamine™ MessengerMAX™ according to the manufacturer's protocol. CFP‐EGFR^L858R^ and YFP‐GRB2 mRNA complexes were transfected at a 3:1 ratio, totalling 600 ng per dish.

### 2.5 MITO staining

Cells were washed once with PBS, stained with 100 µM MitoTracker Deep Red 633, and incubated at 37°C in a 5% CO_2_ incubator for 20 min according to the manufacturer's protocol. Cells were then washed three times with PBS and imaged by fluorescence microscopy.

### 2.6 Live‐cell FRET microscopic imaging

FRET microscopy was performed using an Apotome 2 widefield microscope (Olympus, Japan) to acquire three‐channel FRET images (DD, DA, AA), BF images, and MITO fluorescence images. The imaging system configuration was described previously.^20^, ^52^

### 2.7 *T* scoring

Quantitative FRET analysis was performed as previously described.^20^, ^52^ Briefly, regions of interests (ROIs) were selected from CFP‐EGFR^L858R^‐enriched areas in three‐channel FRET images. The donor‐centric FRET efficiency (*E_D_*) and acceptor‐donor ratio (*R_C_*) were calculated for each ROI. To generate *E_D_*–*R_C_* binding curves, *E_D_* values were binned according to *R_C_* concentration ratios. Mean *E_D_* values for each bin were plotted against *R_C_*, and the resulting curves were fitted using Origin 64 software. Formulas employed were previously published.^20^, ^52^ For each drug‐treated ROI, *T_i_* was calculated using the following equation (Equation 1):

(1) $$T_{i}=x_{i}^{drug}\;-\bar{x^{control}}$$

where $x_{i}^{drug}$ is the *E_D_* of the *i*th drug‐treated ROI, and $\bar{x^{control}}$ represents the mean *E_D_* of control ROIs. The final *T*‐score was defined as the mean *T_i_* across all drug‐treated ROIs and calculated as:

(2) $$T=\frac{1}{n}\sum_{i=1}^{n}T_{i}$$

where *n* denotes the total number of drug‑treated ROIs.

### 2.8 *P* scoring

Single‐cell masks were generated from BF images and MITO fluorescence images using the Cellpose model. CellProfiler^53^ was then used to extract BF features (AreaShape, Texture) and MITO features (AreaShape, Texture, Neighbours, RadialDistribution, Granularity, Intensity) from the single‐cell phenotype regions identified by these masks. Linear discriminant analysis (LDA) was performed to calculate the phenotype‐based (*P*) score, combining both categories of single‐cell features. For each drug‐treated group, data from treated cells and matched control cells were split into training and test sets (ratio 7:3) for LDA model training. For each drug‐treated cell (*i*) in the test set, the phenotype change score (*P_i_*) was computed from the LDA output probabilities as follows:

(3) $$P_{i}=\mathrm{LDA}\left(y\;=\mathrm{drug}|x_{d,i}^{\mathrm{test}}\right)\;-\;\bar{\mathrm{LDA}\left(y\;=\mathrm{drug}|x_{c}^{\mathrm{test}}\right)}$$

Here, $\mathrm{LDA}(y\;=\mathrm{drug}|x_{d,i}^{\mathrm{test}})$ represents the probability from the LDA model that the *i*th drug‑treated test cell is classified as drug‐treated, and $\bar{\mathrm{LDA}(y\;=\mathrm{drug}|x_{c}^{\mathrm{test}})}$ is the average LDA probability of control test cells classified as drug‐treated. The final *P‐*score is the mean *P_i_* across all drug‐treated cells:

(4) $$P=\frac{1}{n}\sum_{i=1}^{n}P_{i}$$

where *n* denotes the number of drug‑treated cells.

### 2.9 *TP* scoring

Using the *T*‐score (Equation 2) and *P*‐score (Equation 4), the integrated *TP*‐score was calculated as follows:

(5) $$TP\;=10\;\times \;[{\left(T\;+1\right)}^{P}-\;1]$$

A higher *TP*‐score, reflecting substantial on‐target activity and notable phenotypic responses, indicates greater drug efficacy according to this evaluation system. For multi‐drug comparisons, *P‐*, *T‐*, and *TP*‐scores were normalised on a scale from 0 to 1, where control = 0 and the highest‐scoring drug = 1.

### 2.10 Cell viability assay

Cell viability was assessed using CCK‐8. Cells were seeded into 96‐well plates, exposed to the indicated drugs, and incubated for 48 h. Subsequently, 100 µL of serum‐free RPMI 1640 medium containing 10% CCK‐8 reagent was added to each well, followed by incubation for 1 h. Absorbance at 450 nm was measured using a microplate reader.

### 2.11 Colony formation assay

Cells (1 × 10^3^ per well) were seeded in 6‐well plates and treated with the indicated drugs on the following day. After 14 days, cells were washed with cold PBS, fixed in 4% paraformaldehyde for 10 min, and stained with crystal violet solution for 10 min.

### 2.12 Wound healing assay

When cultures in 6‐well plates reached approximately 90% confluence, a scratch was made using a 200 µL pipette tip. Microscopy images of the scratch areas were acquired at 0 and 24 h post‐treatment, and scratch closure was quantified using Fiji ImageJ.

### 2.13 Generation of EGFR knockout (KO) bulk populations

The lentiCRISPR v2 system was used to introduce EGFR‐directed CRISPR/Cas9 into H3255 cells. The vector carried either an EGFR‐targeting sgRNA (5′‐GTCCACGCTGGCCATCACGT‐3′) or the non‐targeting control sg‐NC (5′‐GGGGTAGGCTAATTACGGA‐3′). For virus production, HEK293T cells received the sgRNA construct along with the packaging plasmids. H3255 cultures were exposed to the collected viral supernatants with polybrene for 6 h, then returned to complete medium. Puromycin‐resistant cells obtained through subsequent selection were propagated together as a non‐clonal population for further experiments.

### 2.14 Western blotting

Protein input was matched across samples for electrophoretic separation by SDS‐PAGE. After transfer onto PVDF, the membranes underwent milk blocking (5% non‐fat milk) and primary antibody incubation (4°C, overnight). Detection involved sequential application of the appropriate HRP‐conjugated secondary antibodies and enhanced chemiluminescence reagents.

### 2.15 Cell proliferation assays after EGFR knockout

For proliferation measurements, 96‐well plates were prepared with approximately 1000 cells in each well and four replicate wells for every group. Two sets of wells were allocated to CCK‐8 analysis: one for the baseline measurement, obtained 8 h after plating (day 0), and the other for day 5. Each well contained 100 µL of culture medium and received 10 µL of CCK‐8 reagent. The plates remained at 37°C for 1 h before recording the optical density at 450 nm. Proliferation was quantified by dividing the background‐corrected day 5 reading by the corresponding day 0 value: (OD_day 5_ − OD_blank_)/ (OD_day 0_ − OD_blank_). In a separate drug‐response experiment, EGFR‐KO bulk and control cultures were plated in 96‐well plates. Following an 8‐h attachment period, the cells received 0.01 µM osimertinib for 48 h. Viability was then determined with the CCK‐8 procedure described above and expressed relative to the vehicle‐treated cells of the same group.

### 2.16 Mouse xenograft experiments

The animal experiments were approved by the Laboratory Animal Welfare and Ethics Committee of Jinan University (Approval no. IACUC‐20250905‐04). Female BALB/c nude mice (4–6 weeks old) received subcutaneous injections into the right axilla containing 1 × 10^7^ stable H3255 cells suspended in 100 µL of serum‐free medium with 50% Matrigel. Tumour size was monitored every 3 days using calliper measurements, and tumour volume was calculated using the formula: Volume = 0.5 × (largest diameter) × (smallest diameter).^2^ Mice were randomised into treatment groups when the mean initial tumour volume reached approximately 100–150 mm^3^. Randomisation was performed to ensure comparable tumour volumes across groups. Three treatment groups were established, with 5 mice per group. Mice received daily oral gavage with vehicle control (saline), GEF (51.4 mg/kg), or OSI (16.4 mg/kg). Tumour volume and body weight were recorded every 3 days during treatment. After 21 days of treatment, mice were euthanised, and xenografts were excised, photographed, and weighed.

### 2.17 Patient consent and sample collection

This study was approved by the Institutional Review Board of the First Affiliated Hospital of Jinan University (KY‐2024‐230) and conducted in accordance with the Declaration of Helsinki. Written informed consent was obtained from all patients. Surgically resected tumour specimens were collected from patients with LUAD at the First Affiliated Hospital of Jinan University.

### 2.18 Processing of surgical tumour tissues and isolation of primary tumour cells

Tumour tissues were washed with PBS containing 10% antibiotics for 5–10 min. Single‐cell suspensions were prepared according to the manufacturer's instructions for the Miltenyi Tumor Dissociation Kit for human samples. EpCAM^+^ cells were subsequently enriched and isolated from the prepared single‐cell suspensions using immunomagnetic positive selection with the EasySep™ Human EpCAM Positive Selection Kit II. Isolated EpCAM^+^ primary tumour cells were seeded into 15‐mm glass dishes for subsequent analyses.

### 2.19 Preparation of mRNA‐based FRET biosensors

mRNA‐based CFP‐EGFR^L858R^ and YFP‐GRB2 constructs were synthesised using the Yeasen T7 High Yield RNA Synthesis Kit. Co‐transcriptional capping was performed using CAP1 GAG, and UTP was replaced with N1‐Me‐Pseudo UTP (m1Ψ) to reduce immunogenicity.

### 2.20 Exome sequencing

Exome sequencing of the P#1 tumour sample was performed by Tsingke Biotechnology (Beijing, China).

### 2.21 Statistical analysis

Except for *E_D_–R_C_* binding curves, which were generated using Origin 64, all statistical analyses and graphical plots were performed using GraphPad Prism 10. Quantitative data are presented as mean ± standard deviation (SD). Comparisons between two groups were conducted using two‐tailed Welch's *t*‐tests. One‐way ANOVA followed by Dunnett's multiple‐comparison test was used for experiments involving more than two independent groups. For body weight change curves and xenograft growth curves, two‐way ANOVA followed by Dunnett's multiple‐comparison test was applied. Statistical significance was defined as follows: *p < .05, **p < .01, ***p < .001.

## 3 RESULTS

### 3.1 Bioinformatic analysis of EGFR‐GRB2 interaction

To evaluate the on‐target activity of EGFR‐TKIs against EGFR, we performed bioinformatic screening of EGFR‐interacting proteins to identify suitable candidates for FRET biosensor development. Upon activation, EGFR recruits multiple adaptor and signalling proteins to initiate downstream signalling cascades involving VAV2, CBL, GRB2, SHC1, PLCG1, NCK1, STAT1, STAT3, and PTK2.^54^

To identify proteins serving as key mediators of EGFR‐related signalling in NSCLC, we examined the relationships between EGFR and these signalling proteins. First, a physical interaction network was constructed using the STRING database with the highest confidence threshold (0.9). Strong physical associations were identified between EGFR and several proteins, including GRB2, CBL, SHC1, PLCG1, STAT3, and PTK2 (Figure 2A). Among these proteins, GRB2 displayed strong physical associations with all examined signalling proteins except STAT3 and STAT5, whereas STAT3 exhibited the fewest associations among EGFR‐interacting proteins (Figure 2A).

**FIGURE 2:**
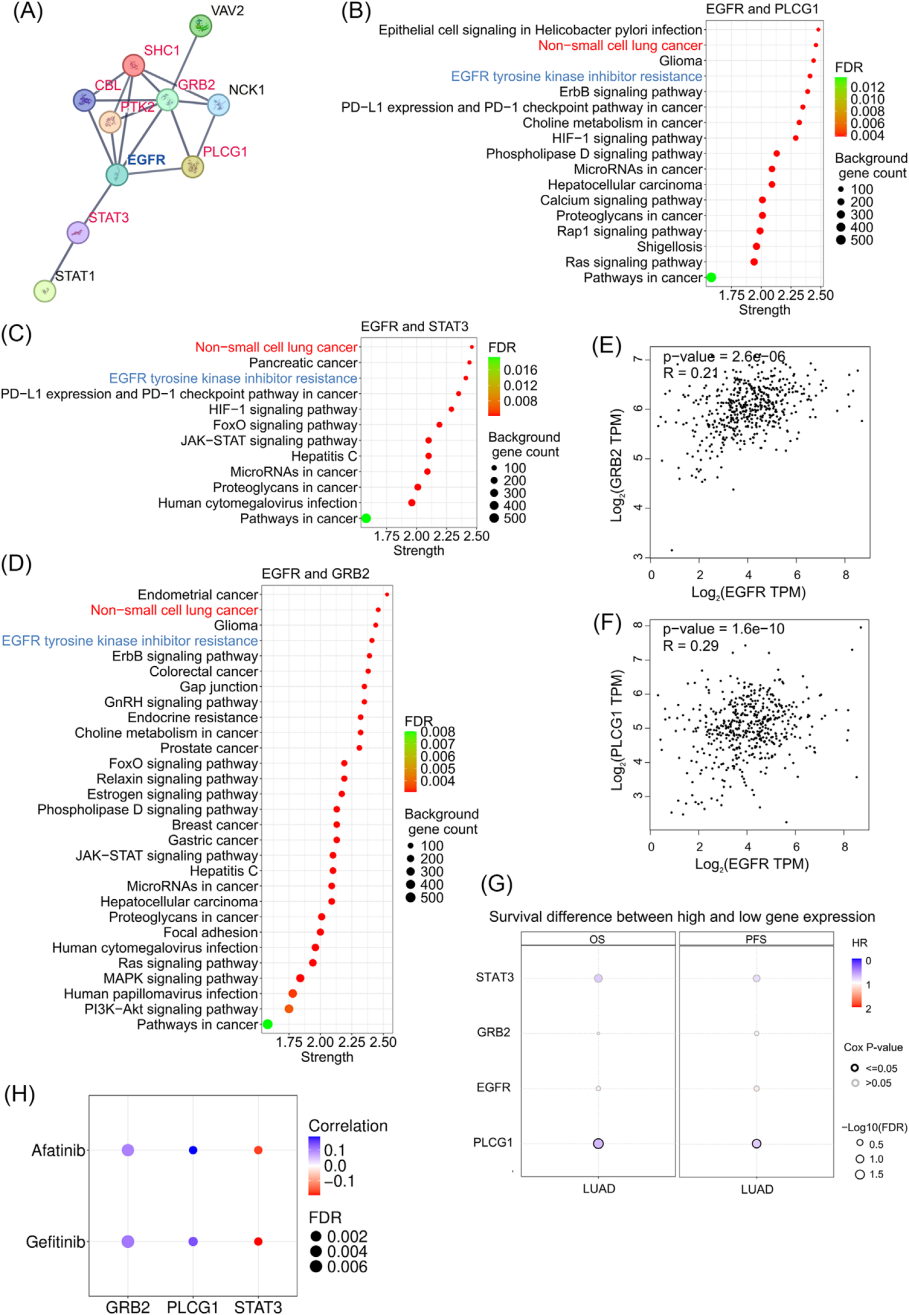
Screening EGFR‐interacting proteins for FRET biosensor development. (A) Physical interaction network generated by STRING, illustrating EGFR interactions with key downstream signalling proteins. Edges indicate physical protein complexes (interaction score > 0.9). (B–D) KEGG pathway enrichment analyses of *EGFR* combined with *PLCG1*, *STAT3*, and *GRB2*, respectively. (E, F) Gene expression correlation analyses between *EGFR* and *GRB2*, *PLCG1*, or *STAT3* in LUAD patients from The Cancer Genome Atlas (TCGA). TPM, transcripts per million. (G) Survival analyses comparing patients with high versus low gene expression. (H) Correlation analyses between gene expression levels and sensitivity to GEF and AFA in pan‐cancer datasets from the Genomics of Drug Sensitivity in Cancer (GDSC). Positive correlations suggest higher gene expression confers drug resistance; negative correlations indicate drug sensitivity.

We next performed KEGG pathway enrichment analysis on *EGFR* and its physically associated proteins, including *GRB2*, *CBL*, *SHC1*, *PLCG1*, *STAT3*, and PTK2. Gene sets containing EGFR together with *GRB2*, *STAT3*, or *PLCG1* were significantly enriched in pathways associated with NSCLC and EGFR‐TKI resistance (Figures 2B–D and S1A–C). Because EGFR mutations in NSCLC occur predominantly in LUAD,^55^ subsequent analyses focused on GRB2, STAT3, and PLCG1 in this subtype.

Gene expression analysis in patients with LUAD showed that *EGFR* expression was positively correlated with *GRB2* and *PLCG1* expression (Figures 2E,F and S1D). However, high *PLCG1* expression was associated with prolonged OS and PFS (Figure 2G), and PLCG1 expression was significantly lower in LUAD tissues than in normal controls (Figure S1E). These findings support the hypothesis that PLCG1 may participate in a negative feedback pathway that suppresses EGFR activation.^54^ Nevertheless, previous studies have also suggested that PLCG1 activation may promote migration and invasion in EGFR‐mutant NSCLC.^56^ The dual and potentially opposing roles of PLCG1 indicate that the EGFR‐PLCG1 interaction cannot reliably predict a consistent biological outcome, limiting its suitability for evaluating the on‐target activity of EGFR‐TKIs. Collectively, these findings suggest that GRB2 acts as a central mediator of EGFR‐driven pro‐tumourigenic signalling in NSCLC.

KEGG pathway enrichment analysis further showed that *EGFR* and *GRB2* were significantly enriched in pathways associated with EGFR‐TKI resistance (Figure 2D). To determine whether *GRB2* expression was associated with EGFR‐TKI resistance, we performed drug sensitivity analysis using GSCA based on the GDSC database. *GRB2* expression was positively correlated with resistance to GEF and AFA across pan‐cancer datasets (Figure 2H). In addition, GRB2 has previously been reported to enhance EGFR‐TKI resistance in NSCLC.^57^

Furthermore, EGFR‐GRB2 colocalisation has been identified as a prognostic biomarker in LUAD.^58^ Importantly, the EGFR‐GRB2 interaction can be quantitatively detected using FRET technology.^45^, ^46^ Therefore, the EGFR‐GRB2 interaction was selected in this study to evaluate the on‐target activity of EGFR‐TKIs.

### 3.2 EGFR^L858R^‐GRB2 interaction‐based *T*‐score of OSI in living H3255 cells

To evaluate whether the EGFR^L858R^‐GRB2 interaction can serve as a marker of EGFR‐TKI activity against EGFR^L858R^ in living cells, we generated CFP‐EGFR^L858R^ and YFP‐GRB2 plasmids. EGFR knockout significantly reduced cell proliferation, supporting an important role for EGFR^L858R^ in promoting proliferation in H3255 cells (Figure S2A and B). Fluorescence microscopy images of H3255 cells expressing CFP‐EGFR^L858R^ alone, YFP‐GRB2 alone, or both proteins together are shown in Figure 3A. In cells expressing CFP‐EGFR^L858R^ alone, fluorescence predominantly localised to the plasma membrane, consistent with the localisation of endogenous EGFR^59^ (Figure 3A, row 1). Cells expressing YFP‐GRB2 alone exhibited a uniform cytoplasmic distribution, consistent with the localisation of endogenous GRB2^60^ (Figure 3A, row 2). In cells co‐expressing CFP‐EGFR^L858R^ and YFP‐GRB2, both proteins co‐localised predominantly at the plasma membrane, suggesting a potential direct interaction (Figure 3A, row 3). To confirm the CFP‐EGFR^L858R^ and YFP‐GRB2 interaction, we performed quantitative FRET imaging analysis. An *E_D_–R_C_* binding curve generated from 95 cells showed a saturable isotherm (Figure 3B), with *E_D_* values approaching saturation at *R_C_* values above 4 (Figure 3B,C), confirming a direct interaction between CFP‐EGFR^L858R^ and YFP‐GRB2. However, because very few data points corresponded to *R_C_* values above 4, limiting practical high‐throughput application, we selected *E_D_* values corresponding to *R_C_* values below 4 for subsequent analyses.

**FIGURE 3:**
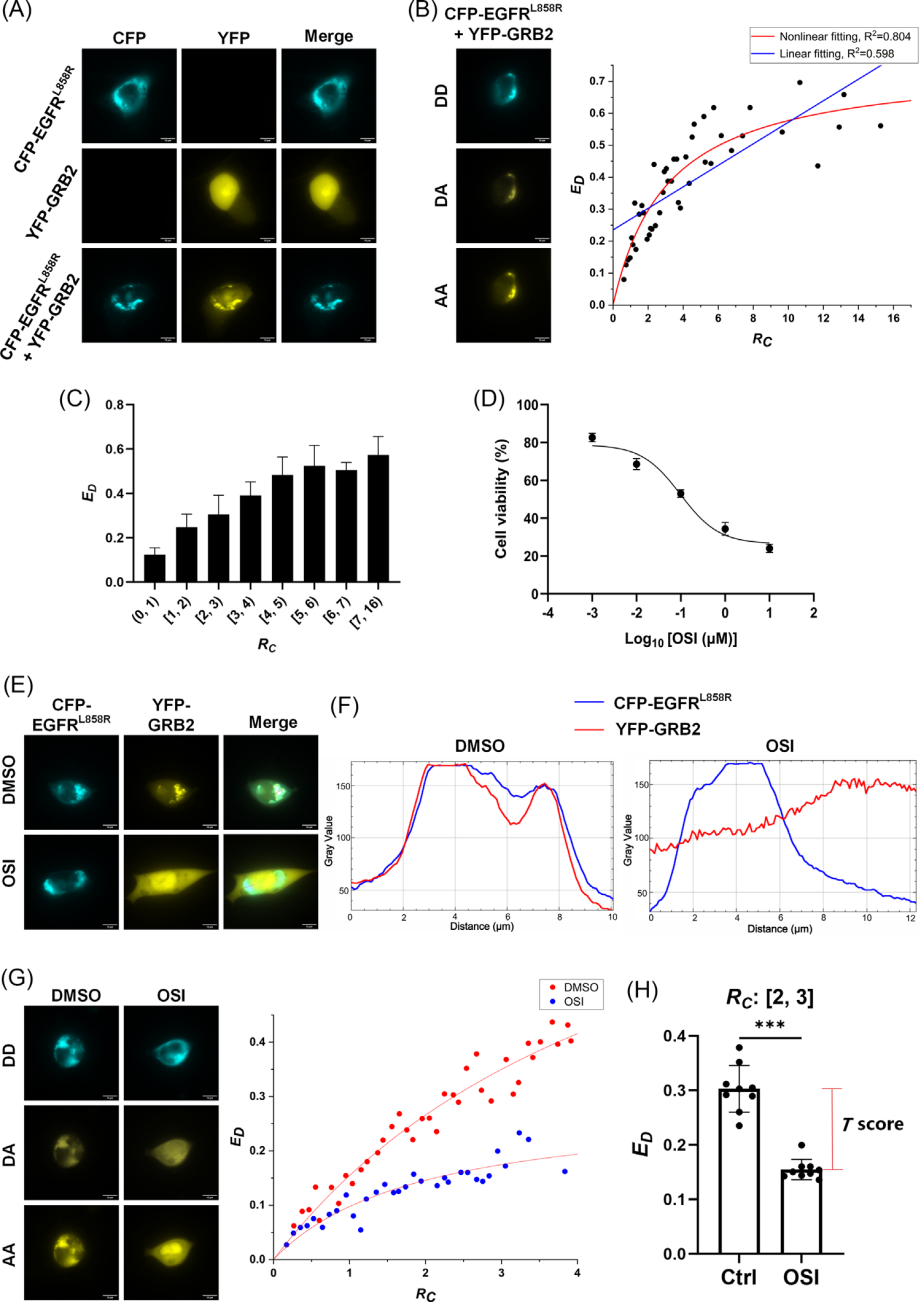
Evaluation of EGFR^L858R^‐GRB2 interactions in living H3255 cells. (A) Representative fluorescence microscopy images of living H3255 cells expressing CFP‐EGFR^L858R^ alone, YFP‐GRB2 alone, or both proteins together. (B) Live‐cell FRET imaging and *E_D_–R_C_* curve analysis of cells co‐expressing CFP‐EGFR^L858R^ and YFP‐GRB2. (C) Histogram illustrating mean *R_C_* values within defined *E_D_* intervals. (D) OSI IC_50_ determination (0.01 µM) by cell viability assay (CCK‐8) after 48 h incubation (*n* = 3). (E, F) Representative fluorescence images and colocalisation analysis of CFP‐EGFR^L858R^ and YFP‐GRB2 in living cells treated for 16 h with 0.0001% DMSO or OSI (0.01 µM). Red lines in merged images indicate regions used for intensity line profiling. (G) Live‐cell FRET analysis following 16 h treatment with DMSO or OSI (0.01 µM). (H) Comparison of mean *E_D_* values within the *R_C_* interval of 2–3 between control and OSI‐treated groups; *T*‐score calculated as the standardised mean difference in *E_D_*. Scale bars = 10 µm.

OSI, a third‐generation EGFR‐TKI, is recommended in the National Comprehensive Cancer Network (NCCN) guidelines as a preferred first‐line treatment option for EGFR L858R‐mutant NSCLC.^61^ To assess OSI's capacity to inhibit the EGFR^L858R^‐GRB2 interaction, cells co‐expressing CFP‐EGFR^L858R^ and YFP‐GRB2 were incubated with DMSO or OSI for 16 h. Initially, the IC_50_ of OSI in these cells was determined to be 0.01 µM using the CCK‐8 assay (Figure 3D); this concentration was then used for subsequent experiments. After 48 h of treatment with 0.01 µM OSI, EGFR‐KO cells exhibited higher relative viability than control cells (Figure S2C), suggesting that EGFR is a key mediator of the inhibitory effect of OSI in H3255 cells. Fluorescence imaging of DMSO‐ and OSI‐treated cells co‐expressing CFP‐EGFR^L858R^ and YFP‐GRB2 is shown in Figure 3E. In DMSO‐treated cells, both CFP‐EGFR^L858R^ and YFP‐GRB2 localised at the plasma membrane and exhibited strong colocalisation (Figure 3E, row 1; Figure 3F). However, in OSI‐treated cells, CFP‐EGFR^L858R^ remained at the membrane, whereas YFP‐GRB2 became diffusely distributed throughout the cytoplasm, resulting in loss of colocalisation (Figure 3E, row 2; Figure 3F). Quantitative FRET analysis demonstrated a significant reduction in the OSI‐treated curve compared to the DMSO‐treated control (Figure 3G), confirming effective inhibition of the EGFR^L858R^‐GRB2 interaction by OSI. Further analysis indicated that *E_D_* values within an *R_C_* range of 2–3 reliably distinguished OSI‐treated cells from controls and provided sufficient data points for quantitative evaluation (Figure 3G,H). Therefore, we constructed a *T*‐score based on the *E_D_* values within this range to quantitatively assess OSI's inhibition of the EGFR^L858R^‐GRB2 interaction (Figure 3H). Specifically, the average *E_D_* values within the *R_C_* range of 2–3 were 0.303 for DMSO‐treated cells and 0.155 for OSI‐treated cells, yielding a *T*‐score of 0.148 for OSI. These results collectively demonstrate that the *T*‐score derived from the EGFR^L858R^‐GRB2 interaction quantitatively reflects the on‐target inhibitory activity of OSI against EGFR^L858R^.

### 3.3 *P*‐score of OSI in living H3255 cells

To evaluate OSI‐induced cellular phenotypic responses, H3255 cells were treated with DMSO or 0.10 µM OSI for 16 h. BF (Figure 4A, list column 1) and MITO (Figure 4A, list column 2) images were acquired, and single‐cell masks were generated (Figure 4A, list column 3) to extract relevant BF and MITO features. Unsupervised dimensionality reduction using UMAP revealed clear separation between DMSO‐ and OSI‐treated cell populations (Figure 4B), indicating significant differences in these features between the two groups.

**FIGURE 4:**
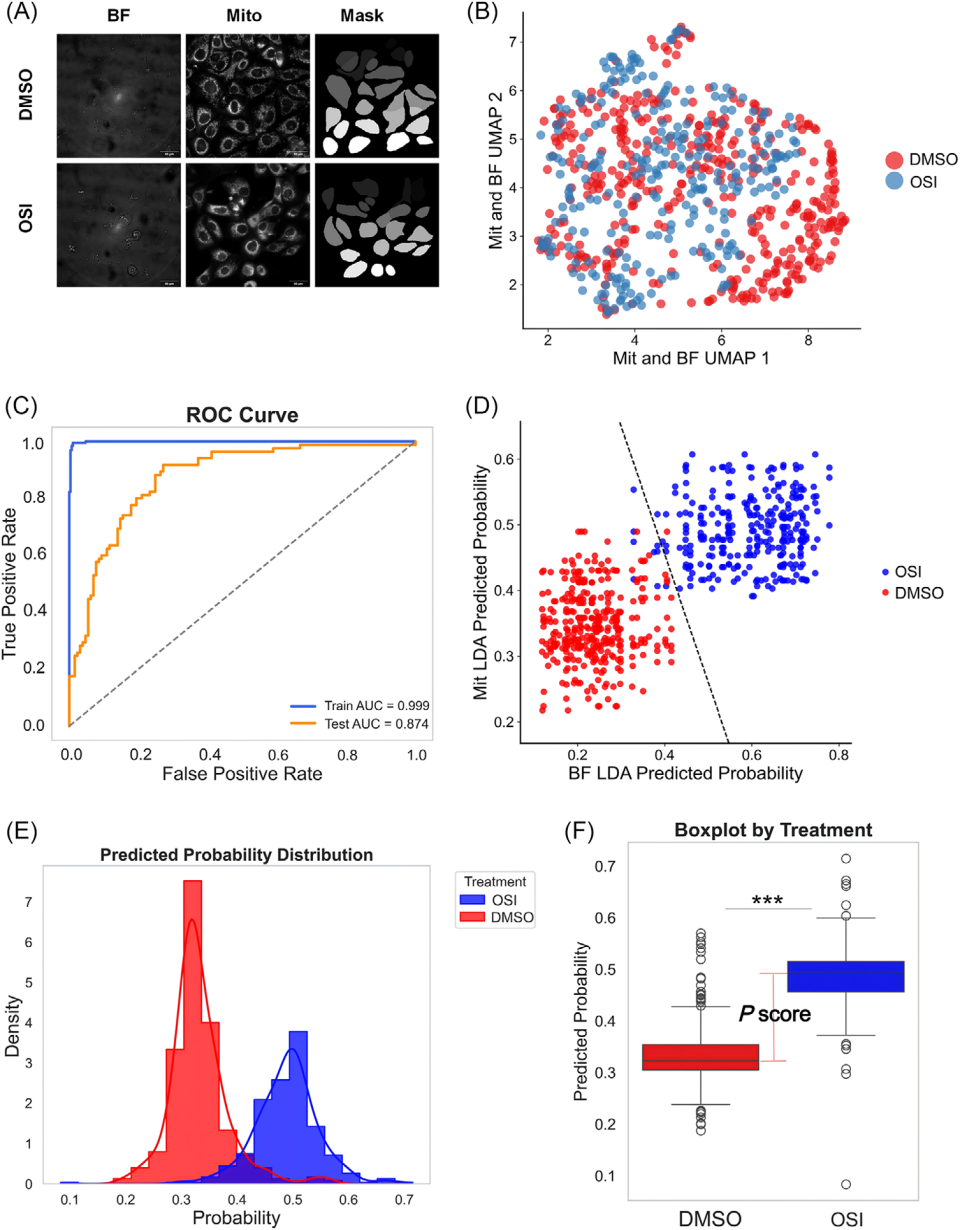
Identification and quantification of phenotypic responses in living H3255 cells. (A) Representative BF and MITO fluorescence images of cells treated for 16 h with 0.0001% DMSO or OSI (0.01 µM), and corresponding single‐cell masks generated using Cellpose. Scale bars = 50 µm. (B) UMAP projection of extracted BF and MITO features. (C) ROC curve performance of the LDA classifier trained on combined BF and MITO features. (D) Scatter plot comparing predicted probabilities derived separately from BF or MITO features. (E) Probability distribution using combined BF and MITO features. (F) Comparison of mean predicted probabilities between DMSO and OSI groups; the *P*‐score calculated as standardised mean difference in predicted probabilities.

To quantify OSI‐induced phenotypic responses, we employed LDA as a supervised classification method. Initially, CellProfiler^53^ was used to extract BF (AreaShape, Texture) and MITO features (AreaShape, Texture, Neighbours, RadialDistribution, Granularity, Intensity) from single‐cell masks. The combined feature dataset was then divided into training and test sets at a ratio of 7:3, and an LDA classifier was trained using the training set. The classifier achieved an area under the receiver operating characteristic curve (AUC) of 0.874 in the test set (Figure 4C), indicating robust discrimination between DMSO‐ and OSI‐treated cells based on integrated BF and MITO features. To assess the added value of feature integration, predicted probabilities were also calculated using BF‐only and MITO‐only features. The combined BF and MITO feature set achieved superior separation compared with either feature set alone (Figure 4D). Using this integrated approach, a statistically significant difference in predicted probability distributions was observed between DMSO‐ and OSI‐treated cells (Figure 4E). Accordingly, the *P*‐score was defined as the mean difference in predicted probabilities between these two groups (Figure 4F). The average predicted probabilities were 0.332 for DMSO‐treated cells and 0.489 for OSI‐treated cells, yielding a *P*‐score of 0.157 for OSI. These findings indicate that the *P*‐score can quantitatively characterise OSI‐induced cellular phenotypic responses.

### 3.4 *TP‐*scores of EGFR‐TKIs in living H3255 cells

According to Equation 5, OSI had a *TP*‐score of 0.219, considerably higher than that of the control (0), demonstrating that the *TP*‐score quantitatively represents OSI's efficacy. We next applied *TP* scoring to predict the efficacy of three EGFR‑TKIs (GEF, AFA, OSI), one ALK‑TKI (ALE), and one chemotherapeutic drug (GEM) in H3255 cells. Cells co‐expressing CFP‐EGFR^L858R^ and YFP‐GRB2 were treated with each drug for 16 h. To approximate maximal in vivo tumour exposure,^62^ drug concentrations were set at their maximum plasma concentrations (*C*_max_) reported in patients receiving recommended clinical doses:^63^ GEF (0.356 µM), AFA (0.052 µM), OSI (0.126 µM), ALE (1.38 µM), and GEM (89.3 µM). Using clinically relevant *C*_max_ concentrations ensures the translational significance of *TP*‐scores.^62^ To confirm that these concentrations were appropriate for *TP* scoring, we first assessed the in vitro antitumour activities of the five drugs at their respective *C*_max_ values in H3255 cells GEF, AFA, OSI, and GEM induced significant cytotoxicity (Figure S3A). Additionally, all five drugs inhibited colony formation (Figure S3B) and reduced cell viability (Figure S3C). GEF, AFA, OSI, and ALE significantly suppressed cell migration (Figure S3D). Collectively, all drugs displayed meaningful in vitro antitumour activity at *C*_max_ concentrations, although ALE demonstrated relatively lower efficacy, as expected given the absence of ALK mutations in H3255 cells. These results confirm the suitability of using *C*_max_ for *TP* scoring in this model.

As shown by live‐cell FRET microscopy (Figure 5A), GEF, AFA, and OSI abolished the colocalisation of CFP‐EGFR^L858R^ and YFP‐GRB2, whereas ALE and GEM did not. Correspondingly, *E_D_*–*R_C_* binding curves for GEF, AFA, and OSI exhibited downward shifts relative to DMSO‐treated controls (Figure 5B). Notably, the curves for AFA and OSI approached linearity (Figure 5B), suggesting near‐complete inhibition of the EGFR^L858R^‐GRB2 interaction by these agents. ALE and GEM, however, produced no significant effects on this interaction (Figure 5B).

**FIGURE 5:**
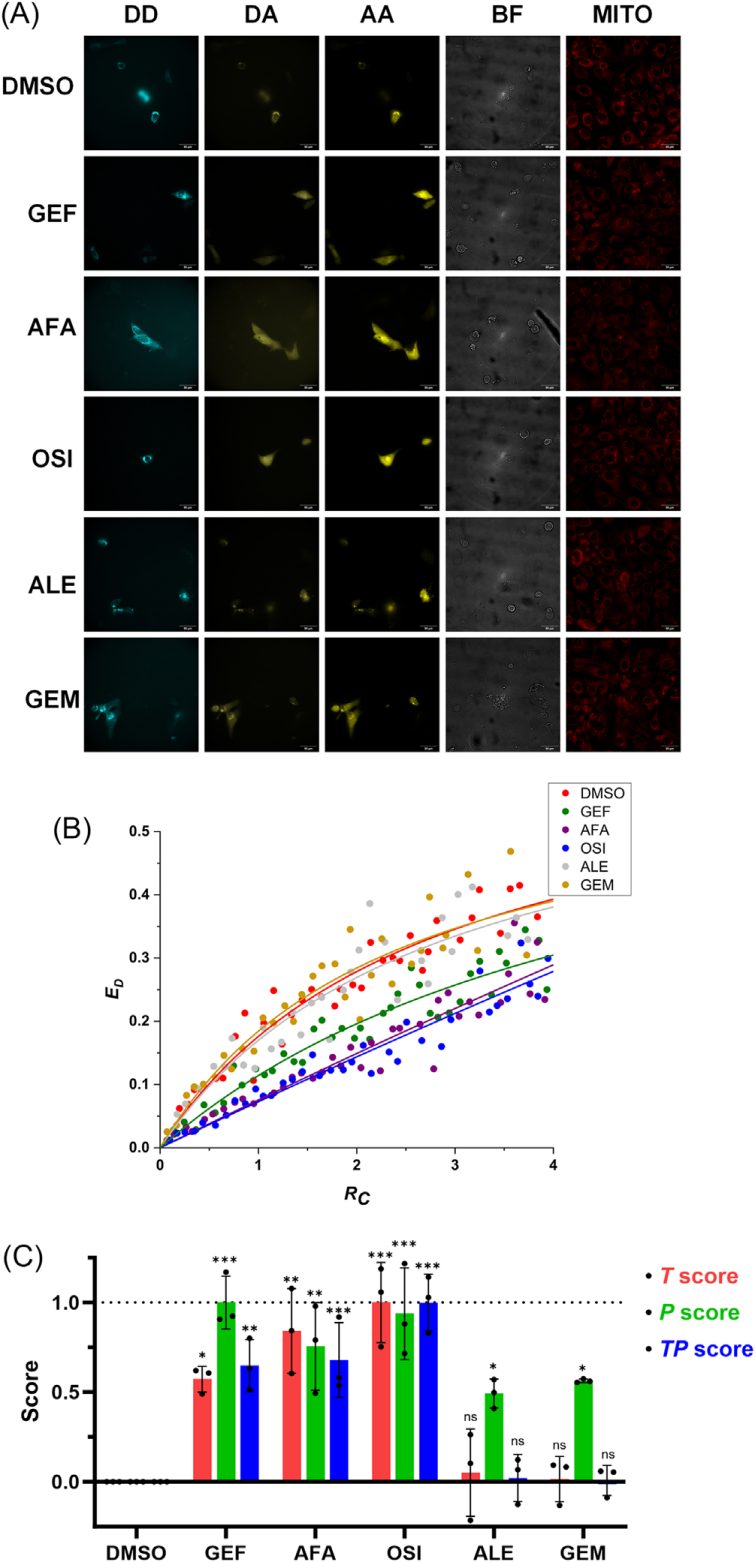
*TP* scoring of EGFR‐TKIs in living H3255 cells. (A) Representative FRET microscopy images of living H3255 cells after 16 h treatment with DMSO or indicated drugs (GEF, AFA, OSI, ALE, GEM), at clinically relevant maximal plasma concentrations (*C*_max_): GEF (0.356 µM), AFA (0.052 µM), OSI (0.126 µM), ALE (1.38 µM), GEM (89.3 µM). Scale bars = 50 µm. (B) Live‐cell FRET profiling (*n* = 3). (C) *TP* scoring results of tested drugs in living H3255 cells (*n* = 3).

Normalised *T‐*, *P‐*, and *TP*‐scores for these five drugs are summarised in Figure 5C. The *T*‐scores ranked highest for OSI (1.0), followed by AFA (0.84), GEF (0.57), ALE (0.05), and GEM (0.02). GEF, AFA, and OSI showed statistically significant *T*‐score differences compared with DMSO, reflecting substantial on‐target inhibition of EGFR^L858R^ by these agents. Regarding *P*‐scores, GEF ranked highest (1.0), followed by OSI (0.94), AFA (0.76), GEM (0.56), and ALE (0.49), with all five drugs producing significant phenotypic responses relative to controls. ALE and GEM displayed relatively low *T*‐scores (∼0.15) and moderate *P*‐scores (∼0.5), consistent with their non‐EGFR‐targeted mechanisms.^64^, ^65^ Finally, *TP*‐scores ranked OSI highest (1.0), followed by AFA (0.68), GEF (0.65), ALE (0.02), and GEM (0.01). GEF, AFA, and OSI demonstrated statistically significant *TP*‐score differences compared with controls, with OSI exhibiting the highest *TP*‐score and GEF the lowest. Thus, the *TP* scoring system predicted greater in vivo antitumour efficacy for OSI relative to GEF in the H3255 cell model.

### 3.5 Validation of *TP*‐scores in H3255 CDX models

To validate the *TP*‐score prediction that OSI has superior in vivo antitumour efficacy compared to GEF in H3255 cells, we conducted a 21‐day monotherapy study using the H3255 CDX mouse model. The doses used for GEF (51.4 mg/kg, oral gavage, QD) and OSI (16.4 mg/kg, oral gavage, QD) were based on recommended clinical dosages^63^ converted to mouse‐equivalent regimens.^66^ Both GEF and OSI significantly inhibited tumour growth, with OSI demonstrating greater antitumour activity than GEF (Figure 6A–C). Neither drug caused significant changes in body weight throughout the treatment period (Figure 6I). Collectively, these findings confirm the *TP*‐score prediction that OSI exhibits greater antitumour efficacy than GEF in vivo.

**FIGURE 6:**
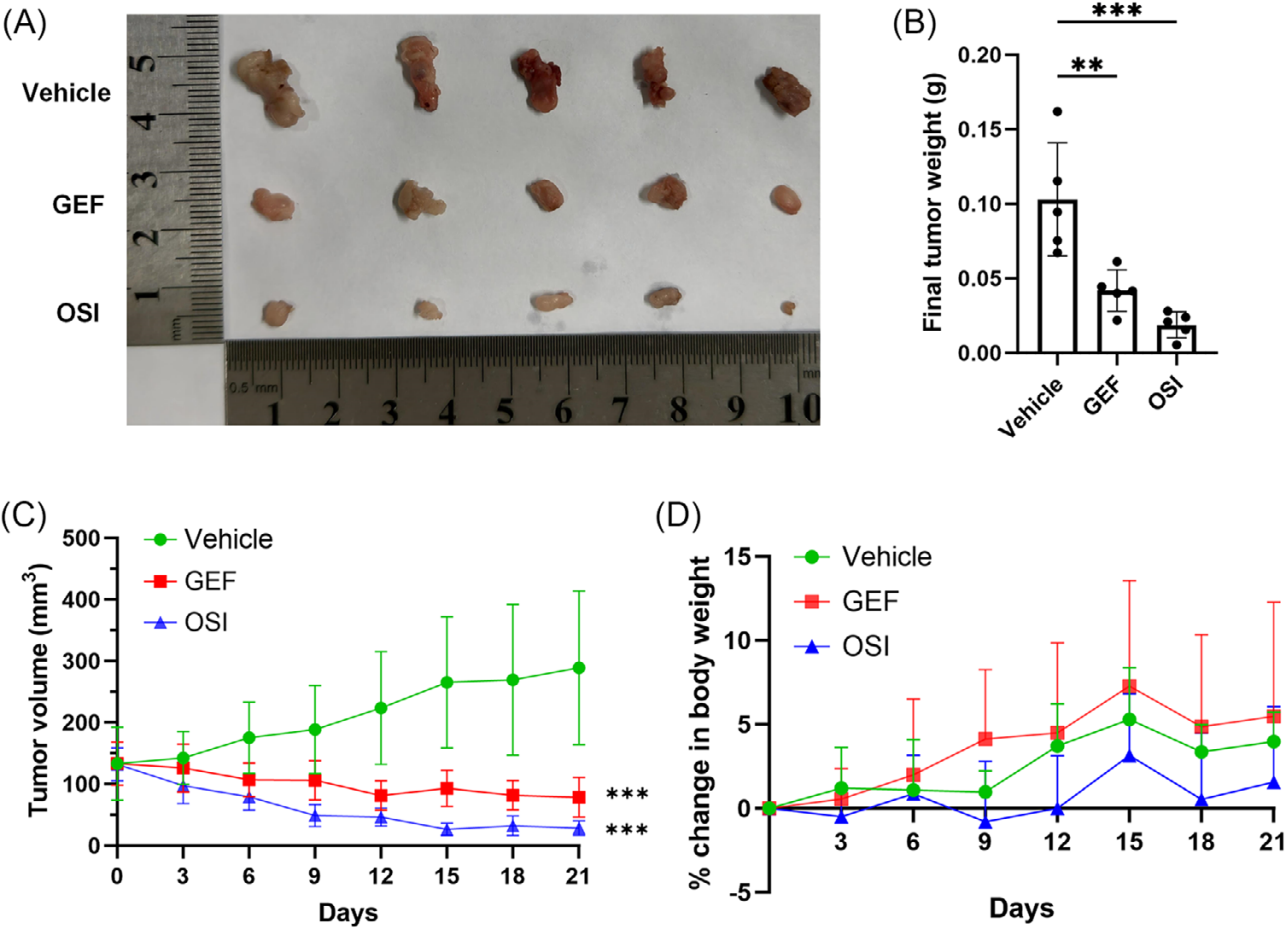
Validation of *TP* scoring in H3255 CDX models. (A) Images of H3255 xenograft tumours excised from BALB/c nude mice after 21 days of indicated treatments (*n* = 5). (B) Tumour weights at day 21 (*n* = 5). (C) Tumour growth curves for H3255 CDX tumours (*n* = 5). (D) Per cent body weight changes in CDX‐bearing mice throughout the treatment period (*n* = 5).

### 3.6 Live‐cell *TP* scoring predicts OSI efficacy in a patient with NSCLC

We next investigated the clinical applicability of live‐cell *TP* scoring. Considering the limited transfection efficiency and high cytotoxicity associated with plasmid‐based delivery in primary cells,^67^ we optimised CFP‐EGFR^L858R^ and YFP‐GRB2 into mRNA‐based constructs.^68^ The structure of the mRNA‐based YFP‐GRB2 construct is illustrated in Figure 7A; its 5’ end was capped with CAP1 GAG, and UTP was substituted with m1Ψ to reduce immunogenicity. The mRNA‐based CFP‐EGFR^L858R^ construct was similarly designed.

**FIGURE 7:**
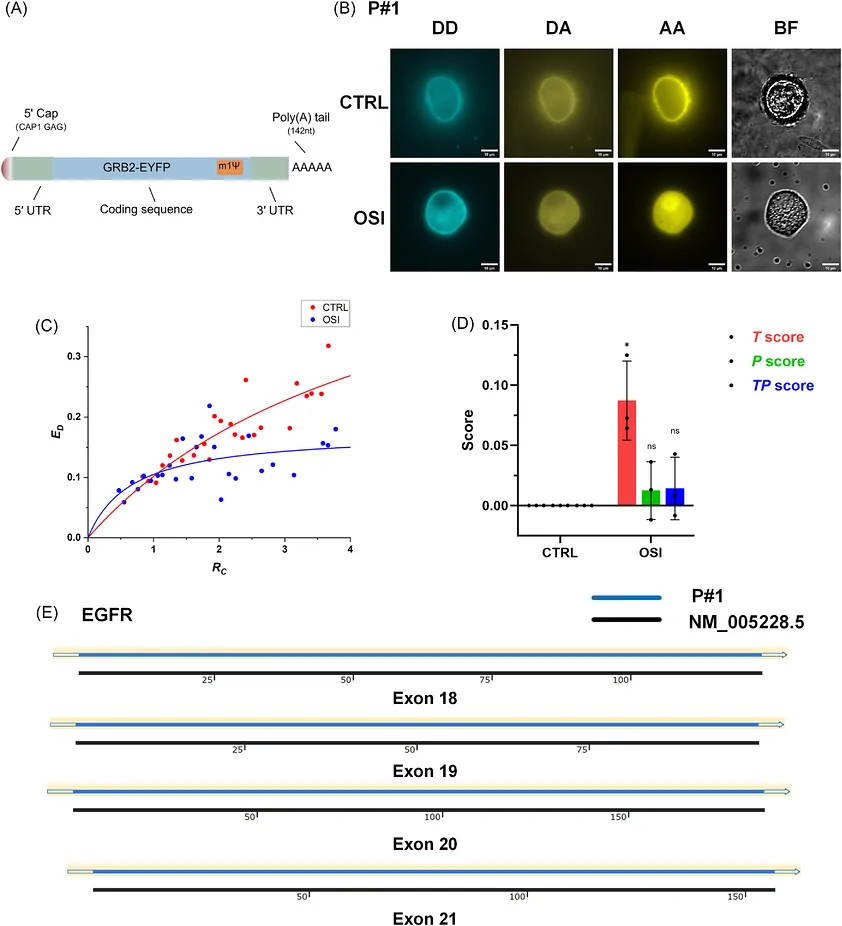
*TP* scoring of OSI in living primary tumour cells from an NSCLC patient. (A) Schematic structure of the mRNA‐based YFP‐GRB2 construct. (B) Representative FRET microscopy images of living tumour cells from patient #1 (P#1) following 16 h treatment with DMSO or OSI (0.126 µM). (C) Live‐cell FRET profiling results (*n* = 3). (D) *TP* scoring results for OSI in living tumour cells from P#1 (*n* = 3). (E) DNA sequence alignment of EGFR exons 18–21 from patient P#1 against the NCBI reference sequence (NM_005228.5).

Patient #1 (P#1) was a 43‐year‐old male with a suspected malignancy in the left lung who underwent surgical resection at our institution. Intraoperative frozen‐section pathology confirmed invasive LUAD; consequently, fresh tumour tissue was collected, and primary tumour cells were isolated. Postoperative pathological evaluation confirmed stage IIIA LUAD (pT1bN2bM0) in the left upper lobe. Immunohistochemical results were EGFR(+), suggesting a potential EGFR mutation. Adjuvant OSI therapy is recommended for patients with resected stage IIIA NSCLC harbouring EGFR L858R or exon 19 deletion mutations.^55^ However, due to financial constraints, the patient did not undergo genetic sequencing to confirm EGFR mutation status. Given the higher prevalence of EGFR L858R mutations relative to exon 19 deletions among male patients with surgically resected stage III LUAD, we conducted EGFR^L858R^‐GRB2 interaction‐based TP scoring to evaluate this patient's potential sensitivity to OSI.

Representative FRET microscopy images of living tumour cells from P#1 after 16 h of treatment with either DMSO or OSI (0.126 µM) are presented in Figure 7B. Due to substantial spontaneous fluorescence interference in the MITO fluorescence channel of primary lung tumour cells, MITO features were excluded from the clinical analysis. OSI treatment eliminated the colocalisation between CFP‐EGFR^L858R^ and YFP‐GRB2 (Figure 7B), resulting in a downward shift of the FRET curves compared to control (Figure 7C). *TP*‐score analysis (Figure 7D) demonstrated a significant increase in the *T*‐score for OSI‐treated cells; however, no significant differences were detected in the *P*‐score or *TP*‐score. These results indicated that while OSI effectively inhibited the EGFR^L858R^‐GRB2 interaction in tumour cells from P#1, it failed to induce meaningful phenotypic responses, suggesting limited therapeutic benefit. This observation raised the possibility that EGFR^L858R^ was not the oncogenic driver mutation in this patient's tumour.

To clarify the low *TP*‐score observed after OSI treatment, EGFR exons 18–21 were subsequently sequenced using the remaining patient‐derived samples, because clinically actionable EGFR mutations typically occur within these exons.^69^ Alignment to the NCBI reference sequence for EGFR (NM_005228.5) confirmed the absence of clinically actionable EGFR mutations in P#1 (Figure 7E). These results explained the observed insensitivity of P#1‐derived tumour cells to OSI. Taken together, live‐cell *TP* scoring effectively predicted OSI insensitivity in this patient with NSCLC, highlighting the potential of this approach for prospectively identifying ineffective treatments.

## 4 DISCUSSION

This study provides preliminary evidence supporting the clinical feasibility of predicting EGFR‐TKI efficacy by integrating the EGFR^L858R^‐GRB2 interaction with cellular phenotype features. The *T*‐score, based on the EGFR^L858R^‐GRB2 interaction, effectively assesses the on‐target activity of EGFR‐TKIs against EGFR^L858R^. The integrated *TP*‐score further incorporated drug‐induced phenotypic responses and provided an overall prediction of EGFR‐TKI efficacy. Consequently, this *TP* scoring approach may support personalised EGFR‐TKI selection for patients with EGFR L858R‐mutant NSCLC.

The agreement between *TP* scoring predictions for GEF and OSI and their efficacy observed in both H3255 CDX models (Figure 6)^70^ and previously untreated patients with EGFR L858R‐mutant advanced NSCLC^71^ highlights the potential utility of *TP* scoring in guiding precision therapy. Although GEF and OSI showed similar cellular phenotypic responses (*P*‐scores) in H3255 cells (Figure 5), OSI exhibited a higher *T*‐score compared to GEF, resulting in a superior *TP*‐score (OSI: 1.0 vs. GEF: 0.65). These findings align with results from the CDX mouse models (Figure 6). While maximal phenotypic inhibition was achievable by both GEF and OSI in vitro due to saturated EGFR pathway blockade.^72^ However, the weaker antitumour activity of GEF in vivo may be attributable, at least in part, to its limited ability to maintain sustained EGFR inhibition as drug concentrations decline.^70^

Single‐cell *TP* scoring can distinguish drug‐resistant from drug‐sensitive subpopulations, facilitating targeted selection of combination therapies in heterogeneous tumours.^3^, ^8^ Approximately 63%–69% of somatic mutations in renal carcinomas exhibit intratumoural regional variability,^73^ and 77% of lung cancer patients show heterogeneous EGFR expression across tumour cells.^74^ Tumour cell heterogeneity typically confers variable sensitivity to monotherapy.^3^ Identifying drug‐resistant subpopulations within predominantly sensitive tumour populations has important clinical relevance, because combination regimens targeting both sensitive and resistant subsets may be more effective than single‐agent therapy.^3^ For instance, Alsaed et al. reported heterogeneous EGFR expression in EGFR‐mutant NSCLC, identifying EGFR‐low cells intrinsically resistant to OSI. However, combining HDAC inhibitors with OSI effectively overcame this resistance.^74^ Similarly, Hata et al. showed that combining an EGFR T790M‐specific inhibitor with an apoptosis‐targeting agent restored sensitivity in cells that had acquired resistance through EGFR T790M mutations.^75^

The *TP* scoring procedure preserves cell viability, which may enhance its clinical practicality. First, the short assay duration of *TP* scoring, approximately 16 h, compares favourably with many functional assays that require days to months.^76^ As a result, cells remain suitable for further assays such as cell viability tests,^76^ SMPC technology,^12^ or LC‐MS/MS analysis.^14^ Combining *TP* scoring with additional functional assay results could further improve the accuracy of personalised treatment guidance. Second, repeated dynamic assessments at multiple time points from the same sample may reduce variability caused by differences in the onset time of drug responses.

The live‐cell *TP* scoring protocol aligns well with clinical workflows. From tumour resection to final analysis, the entire assay can be completed within 48 h. Primary tumour cells generally adhere within 24 h; combined with the subsequent 24‐h *TP* scoring step, the entire process comfortably fits within the 10‐day turnaround time recommended by clinical guidelines.^77^ Minimising ex vivo culture duration is advantageous, as even short‐term (8 days) in vitro culture can alter the therapeutic sensitivity of primary tumour cells.^78^ Additionally, employing clinically relevant maximal plasma drug concentrations (*C*_max_) in live‐cell *TP* scoring ensures the translational validity of the observed responses by closely simulating maximal in vivo tumour exposure.^62^, ^63^

Further improvements in *P*‐score sensitivity may be achieved by incorporating unsupervised deep‐learning approaches for single‐cell phenotypic analysis. The low OSI *P*‐score observed in P#1 was consistent with the absence of actionable EGFR mutations. However, the current supervised CellProfiler‐ and LDA‐based analysis may have limited sensitivity for detecting subtle drug‐induced phenotypic changes. Recent unsupervised deep‐learning methods, such as MorphoGenie^79^ and PhenoProfiler,^80^ have shown superior phenotypic discrimination compared with conventional machine‐learning approaches. In evaluations using a large‐scale dataset comprising nearly 400 000 high‐content images and more than 8 million single‐cell images, PhenoProfiler consistently outperformed existing methods, with improvements in accuracy and robustness of up to 20%.^80^

Three‐dimensional (3D) models represent an ideal platform for future clinical translation of the *TP* scoring. The two‐dimensional (2D) cultures of patient‐derived tumour cells used in this study do not fully recapitulate the in vivo tumour microenvironment.^76^ Previous applications of FRET and HCS in 3D models support the feasibility of extending our platform to 3D models.^81^, ^82^ Anand et al. used a FRET‐based biosensor to monitor paclitaxel‐induced apoptosis in a 3D breast tumour model,^82^ and Koedoot et al. developed an ex vivo, HCS‐based 3D microtumour platform to predict changes in target lesions and PFS in response to platinum‐based chemotherapy in patients with ovarian cancer.^81^

In summary, our findings support the feasibility of integrating the EGFR^L858R^‐GRB2 interaction and cellular phenotype assessment through live‐cell FRET microscopy as a predictive approach for evaluating EGFR‐TKI efficacy. Further studies are required to establish the capability of *TP* scoring in discriminating heterogeneous tumour cell populations, as well as its practical applicability and predictive accuracy in clinical settings. Ultimately, we anticipate that *TP* scoring can contribute meaningfully to personalised treatment decisions for cancer patients, thus minimising unnecessary exposure to ineffective therapies.

## AUTHOR CONTRIBUTIONS

XL, TC, XW and SW conceived and designed the study. LD, SH and CP extracted and analysed the data. PY and SW provided the clinical samples. SL and BS interpreted the data. XL drafted the manuscript. All authors were involved in revising and approving the manuscript.

## CONFLICT OF INTEREST STATEMENT

The authors declare no conflicts of interest.

## ETHICS STATEMENT

The study protocol for human sample collection was reviewed and approved by the Institutional Review Board of the First Affiliated Hospital of Jinan University (KY‐2024‐230) and complied with the Declaration of Helsinki. Written informed consent was obtained from all participants. All animal experiments were approved by the Laboratory Animal Welfare and Ethics Committee of Jinan University (Approval no. IACUC‑20250905‑04).

## CONSENT FOR PUBLICATION

Consent for the publication of patients’ clinical data was obtained by the authors.

## Supporting information



FIGURE S1. (A–C) KEGG pathway enrichment analyses of EGFR combined with CBL, PTK2, and SHC1, respectively. (D) Gene expression correlation analysis between EGFR and STAT3 in LUAD patients. (E) Comparison of EGFR, PLCG1, STAT3, and GRB2 expression levels in LUAD tumour tissues versus paired normal tissues (T vs. N). LUAD samples were obtained from TCGA; paired normal samples were obtained from TCGA and Genotype‐Tissue Expression (GTEx) databases.

FIGURE S2. (A) Representative Western blots and quantification of EGFR expression in control and EGFR‐knockout (KO) bulk cells (*n* = 3). EGFR levels were normalised to β‐actin and expressed relative to control cells. (B) Cell proliferation assessed as the ratio of CCK‐8 absorbance at day 5 to that at day 0 (*n* = 3). (C) Relative cell viability after 48 h of treatment with OSI (0.01 µM), normalised to the corresponding DMSO‐treated control (*n* = 4).

FIGURE S3. (A) Drug‐induced cytotoxicity evaluated by crystal violet staining after 72 h incubation. (B) Colony formation assays assessing clonogenic potential. (C) Cell viability determined using the CCK‐8 assay after 48 h incubation (*n* = 3). (D, E) Cell migration analysed via wound‐healing assays with quantitative measurements (*n* = 3).

## Data Availability

All data are available from the corresponding author upon reasonable request.
